# Expanding the Distribution of *Prosthechea jauana* (Orchidaceae) in the Pantepui and Highlighting the Urgent Need for Conservation Strategies in the Region in Face of Climate Change

**DOI:** 10.3390/plants13020222

**Published:** 2024-01-13

**Authors:** Tiago L. Vieira, Rafael G. Barbosa-Silva, André L. Acosta, Cássio van den Berg

**Affiliations:** 1 Harvard University Herbaria, Department of Organismic and Evolutionary Biology, Harvard University, 22 Divinity Avenue, Cambridge, MA 02138, USA; 2 Biodiversity and Ecosystem Services, Instituto Tecnológico Vale Desenvolvimento Sustentável, Belém 66055-090, Pará, Brazil; rafa.g29@gmail.com (R.G.B.-S.); andreluisacosta@gmail.com (A.L.A.); 3 Coordenacão Botânica, Museu Paraense Emílio Goeldi, Belém 66077-830, Pará, Brazil; 4 Departamento de Ciências Biológicas, Universidade Estadual de Feira de Santana, Feira de Santana 44036-246, Bahia, Brazil; vcassio@uefs.br

**Keywords:** Amazonia, Anthropocene, Guiana Shield, Neotropics, species distribution, tepui

## Abstract

*Prosthechea jauana* has been recognized as an orchid species endemic to the Venezuelan tepui. The first record of *P. jauana* in Brazil is presented here, also from a tepui in the Southern phytogeographical district of Pantepui in the Serra do Aracá, at the northern border of the Amazonas state. A detailed morphological description and images of the specimen are presented, as well as an updated distribution map, preliminary conservation status assessment, and taxonomic notes about the species. In addition, we provide species’ distribution models for *P. jauana* based on current and future bioclimatic data. Future projections suggest that the geographic distribution of *P. jauana* will likely be severely affected, with ~79% of its suitable habitat being reduced by 2041–2060 and ~92% by 2061–2080. *Prosthechea jauana* could represent a flag species and an example of how climate change may affect the endemic Pantepui flora.

## 1. Introduction

*Prosthechea jauana* (Carnevali & I. Ramírez) W.E.Higgins is an orchid species that has been recognized, to date, as being endemic to the Venezuelan Pantepui [1]. The Pantepui is a biogeographical province with an assemblage of orographic ecosystems on the summit of the flat-topped mountains across the Guiana Shield highlands (i.e., the tepuis), consisting of a true sky island archipelago surrounded by lowland rainforest, and harbors a remarkably rich and endemic biodiversity [2]. Orchidaceae is the richest family in the Pantepui vascular flora, corresponding to ~10% (258 species) of the total flora. However, only 20% of the listed orchid species are endemic to the Pantepui [3], and *Prosthechea jauana* is one of these.

The species was originally described based on materials from the tepuis of Meseta de Jaua, in Venezuela [1]. It belongs to a group of morphologically related species including *Prosthechea aemula* (Lindl.) W.E.Higgins, *P. chimborazoensis* (Schltr.) W.E.Higgins, *P. fragrans* (Sw.) W.E.Higgins, *P. sima* (Dressler) W.E.Higgins, and *P. venezuelana* (Schltr.) W.E.Higgins, also known as the *P. fragrans* complex [4]. The distinguishing morphological features of this species group, when compared to other species of *Prosthechea* Knowles & Westcot., are the one-leaved pseudobulbs, whitish flowers with acuminate sepals and petals, and convex lip covered by purple lines along its extension.

**Figure 1:**
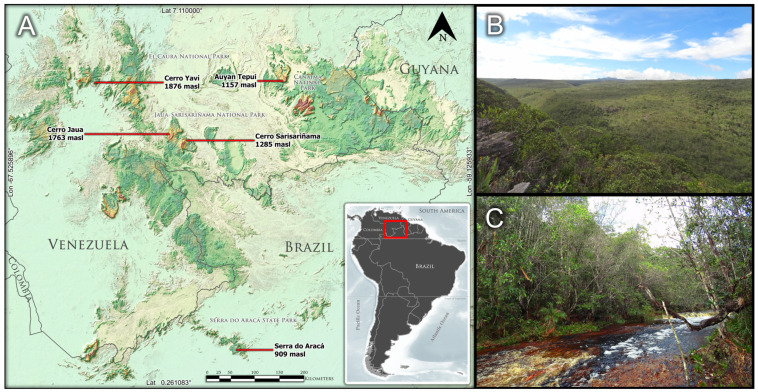
Distribution and occurrence of *Prosthechea jauana.* (**A**). Distribution map of *P. jauana*. The elevation of known records is indicated according to the tepui of occurrence. High elevations in reddish colors. Vouchers/literature records: Ayuán tepui (Dunsterville A99/A), Cerro Jaua (Steyermark et al. 109478), Cerro Sarisariñama (Steyermark et al. 109159), Cerro Yaví (Michelangeli 166), and Serra do Aracá (Labiak et al. 5690, new record in Brazil). (**B**). Montane forest formations on higher slopes of the Serra do Aracá. (**C**). Forest formations along water drainage areas in the Serra do Aracá.

Until now, *Prosthechea jauana* had only been found in the Venezuelan tepuis, including the two neighboring mountains of the Meseta de Jaua formation [Cerro Jaua (type locality) and Cerro Sarisariñama], in the Jaua-Duida district, state of Bolívar, and the Cerro Yaví, in the Western district, state of Amazonas [5]. However, after an extensive revision of materials for a broader taxonomic study of the Brazilian species of *Prosthechea* [6], a collection of the species from Serra do Aracá (also a tepui), in the Brazilian state of Amazonas, was recognized (Figure 1). The material was collected during a field expedition for the Vascular Flora of Serra do Aracá project [7] and had been misidentified as *P. fragrans*.

Accelerated biodiversity loss due to climate change is an ongoing and urgent issue, which is ultimately related to human activities in the rapidly changing world of the Anthropogenic era (i.e., Anthropocene) [8,9,10]. In this context, biological collections play a key role in providing species records with a spatiotemporal dimension, and the public availability of data has been rising exponentially after digitization efforts in the last few decades, which turns these collections into a powerful source of data for conservation planning and predicting the patterns and processes in future biodiversity faced by a changing planet [11].

Despite the limited empirical evidence for climate-driven global plant extinctions to date, studies have been pointing towards shifts in vegetation distribution, especially in montane environments where elevation gradients tend to reflect very particular ecological niches and, consequently, community assemblages [12,13,14]. Among global mountain ecosystems threatened by climate change, the Pantepui region deserves special attention. Recent studies with an emphasis on vascular plants have been quantitatively estimating potential extinction risks induced by climate change in the region [14,15,16,17,18]. The projected future scenarios are alarming, with temperature elevation and a decrease in precipitation rates leading to habitat loss. Estimates suggest a potential habitat loss of 22–46% for the vascular plant species endemic to the region [14]. Differently from other cone-shaped mountains, the Pantepui present as flat-topped mountains, which directly restrict species’ upward migration following temperature elevation and ecological niche shift. The lack of potential climatic refuges has profound implications for plant populations, especially those of rare and endemic species. Many species are experiencing declines in population size and distribution range as they struggle to adapt to changing environmental conditions.

Herein, we present this southward expansion of *Prosthechea jauana* geographic distribution in the Pantepui region, representing a new record in the Brazilian flora. A morphological description of the material, images of the specimens with floral dissection details, taxonomic notes, an updated distribution map, and a preliminary conservation status assessment are provided. Additionally, we perform species distribution modeling (SDM) using bioclimatic variables, comparing current conditions and ecologic preferences against future scenario projections to discuss conservation implications.

## 2. Results

### 2.1. Taxonomy

**Figure 2:**
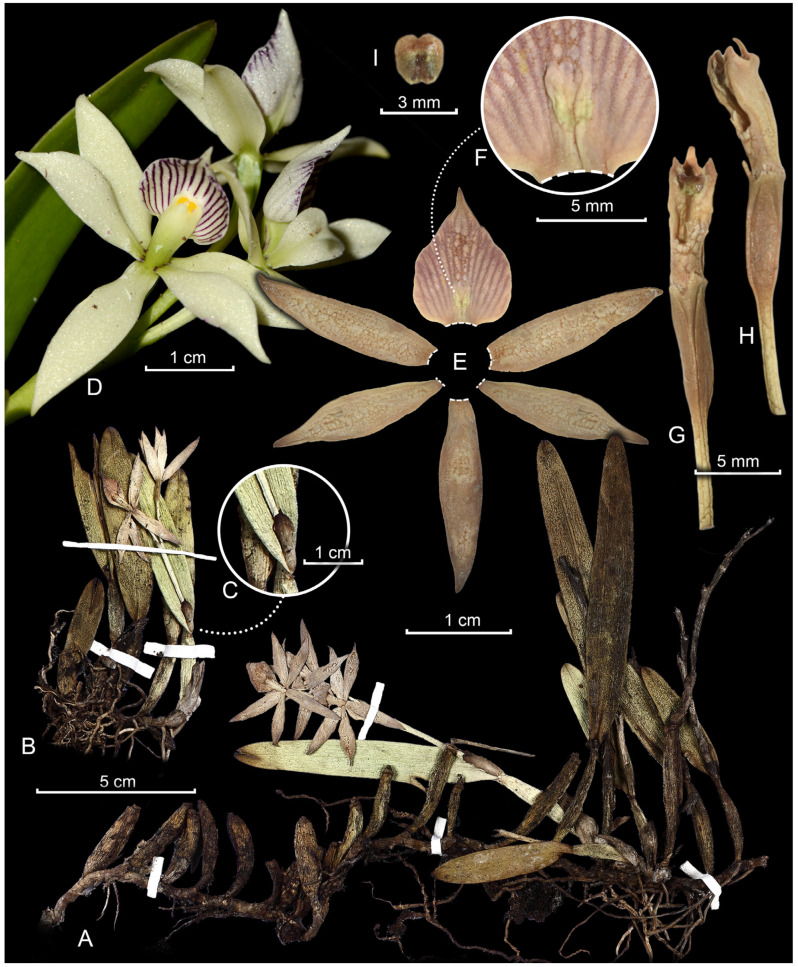
*Prosthechea jauana*, a new record in the Brazilian flora. Digital plate based on the collection Labiak et al. 5690 (RB). (**A**,**B**). Habit of the two specimens present on the sheet. (**C**). Spathe detail. (**D**). Inflorescence detail, highlighting flower colors (colored photography of the specimen presented in (**A**)). (**E**). Perianth dissection (flower picked of specimen (**B**)). (**F**). Callus detail. (**G**,**H**). Ventral and lateral view of the column and ovary with pedicel, respectively. (**I**). Anther cap detail. Edited by T.L.V.

*Prosthechea jauana* (Carnevali & I. Ramírez) W.E.Higgins, Phytologia 82(5): 378. 1997 [1998]. ≡ *Encyclia jauana* Carnevali & I. Ramírez, Lindleyana 9(1): 67. 1994. ≡ *Anacheilium jauanum* (Carnevali & I. Ramírez) Withner & P.A.Harding, Cattleyas and Relatives: Debatable Epidendrums: 96. 2004. TYPE: Venezuela. Bolívar: Meseta de Jaua, summit of Cerro Jaua, portion SW, 1750–1800 m, 22–28 February 1974, J.A. Steyermark, C. Espinoza & C. Brewer-Carias 109478 (Holotype: VEN!). Figure 2.

Epiphytic herb, rhizomatous, 5.7–16.5 cm tall. Rhizome 0.6–1.3 cm long. Pseudobulbs 1-leaved, fusiform, 1.5–4.5 × 0.4–1.0 cm, slightly laterally compressed. Leaves coriaceous, narrowly oblong to narrowly elliptic, 3.2–10.5 × 1.0–2.2 cm, obtuse. Inflorescence growing from mature pseudobulb, 6.0–8.3 cm long, 2–5-flowered; spathe 1.1–2.5 cm long. Flowers non-resupinate, somewhat fleshy; pedicellate ovary 1.0–1.5 cm long; sepals creamy white, dorsal narrowly elliptic, 1.9–2.1 × 0.4–0.6 cm, acute, laterals lanceolate to narrowly elliptic, 1.8–2.1 × 0.4–0.5 cm, acute; petals creamy white, elliptic, 1.7–1.9 × 0.4–0.6 cm, acuminate. Labellum entire, clawed, adnate to the column for ca. 3 mm, the free portion white with longitudinal vinaceous lines throughout its length, concave in its natural position, ovate when flattened, 1.2–1.5 × 1.1–1.3 cm, acuminate; callus pad-like, oblong, ca. 5.0 × 2.0 mm, depressed at the apex, glabrescent. Column subclavate, 8.0–8.5 × 3.0–4.0 mm; clinandrium 3-toothed, the teeth are subequal, ca. 1 mm long, the midtooth ligulate and bearing a fleshy knob-like dorsal appendage; anther capsule subglobose, ca. 2.0 × 2.0 mm; pollinia, laterally flattened. Capsule not seen.

**Specimens examined: Brazil**. Amazonas: Barcelos, Parque Estadual da Serra do Aracá, Eldorado, waterfalls trail towards the summit, 1000–1150 m, 22 April 2014, Labiak et al. 5690 (RB). **Venezuela**. Amazonas: Cerro Yaví, 2100 m, 24 February 1995, Michelangeli 166 (BH!, VEN!). Bolívar: Meseta de Jaua, Cerro Sarisariñama, summit, 1400 m, 16–18 February 1974, Steyermark et al. 109159 (VEN!); ibid., Cerro Sarisariñama, February 1974, fl. in cult. May 1974, Dunsterville and Dunsterville 1316 (AMES!, line drawing); Meseta de Jaua, Cerro Jaua, summit of cerro, in an area near a tributary of the Marajano River, February 1974, fl. in cult. April 1974, Dunsterville and Dunsterville s.n. (VEN 96407!).

**Etymology:** The epithet refers to the species type’s locality, which is the Meseta de Jaua, in Bolívar State, Venezuela.

**Distribution and habitat:** Endemic to the Pantepui in southern Venezuela and the northern border of Brazil. So far, *Prosthechea jauana* has been found on Cerro Yaví, Meseta de Jaua, and Serra do Aracá. It occurs in montane forest formations on higher slopes (above 1000 m) and the shrubby vegetation on the flat tops of the tepuis, usually as an epiphyte and occasionally as a litophyte. The specimens’ labels usually refer to the forophytes as small shrubs, and only a paratype from Cerro Sarisariñama specifies the forophytes as small trees of *Bonnetia* sp.

**Phenology:** Specimens with flowers were collected in February and April.

**Conservation status:** Based on the known distribution records of the species, its estimated EOO and AOO values are 101,148 km^2^ and 20 km^2^, respectively, which would place the species into the Endangered (EN) category under criteria B2. However, all known occurrence locations are within protected areas in the respective countries, including the Serra do Aracá State Park in Brazil, the Caura National Park, Canaima National Park, and the Cerro Yaví Natural Monument in Venezuela. Nevertheless, the Serra do Aracá region has been threatened in the past, and there is still pressure from mining activities in the area [7]. Urgent actions are needed to address the damage caused by the mining arc in the Pantepui region, which has impacted areas such as the Canaima National Park [19,20], close to some of the known *P. jauana* populations. In addition, the results of our distribution modeling analysis toward future bioclimatic conditions suggest potential threats from climate change to populations across the tepuis (see Species Distribution Modeling topic ahead in the results section).

Considering that there is no empiric data available about population reduction, generation length, decline in habitat quality, or populational fluctuation to assess the species against most of the required criteria and subcriteria (A, B, C and E), but given the information on plausible future threats, and that the current known occurrences are ≤ 5, we are assessing the species as Vulnerable (VU) following criteria D2. In this case, future threats can drive the taxon into a higher risk category, which would lead to moving the species into more critical categories.

**Taxonomic Notes:** *Prosthechea jauana* is related to the *P. fragrans* complex [4], and is morphologically similar to *P. aemula* and *P. fragrans* itself, especially the flowers. However, the three species may be readily distinguished based on vegetative traits, ecological aspects, and geographic distribution. *Prosthechea aemula* is widely distributed across the Neotropical region, occurring in warm lowland habitats such as rainforests (usually from sea-level to 600 m), and presents as remarkable character its inflorescences always developing from still immature pseudobulbs (the new sympodium blooms while yet a shoot, generally). Conversely, the inflorescences of *P. fragrans* and *P. jauana* develop from completely mature pseudobulbs. Regarding geographic distribution, *P. fragrans* is also a warm lowland habitat species, distributed throughout the West Indies and Central America [21], whereas *P. jauana* is endemic to the Pantepui province (above 1000 m) in Brazil and Venezuela, on the western Guiana Shield. Furthermore, *P. fragrans* plants are robust and large, having large fusiform pseudobulbs (3.5–15.0 cm long) and long spathes (up to 5 cm long), whereas *P. jauana* plants are smaller, with ovoid to fusiform, shorter pseudobulbs (2.2–4.5 cm long) and shorter spathes (up to 2.5 cm long). Additionally, identification keys for this set of species may be found in previous taxonomic contributions [1,5], where the authors also mention further cryptic floral traits which separate *P. jauana* from *P. aemula* and *P. fragrans*, such as the stigmatic surface, which is ovate-cordate in the former and transversally pandurate in the other two.

### 2.2. Species Distribution Modeling

**Figure 3:**
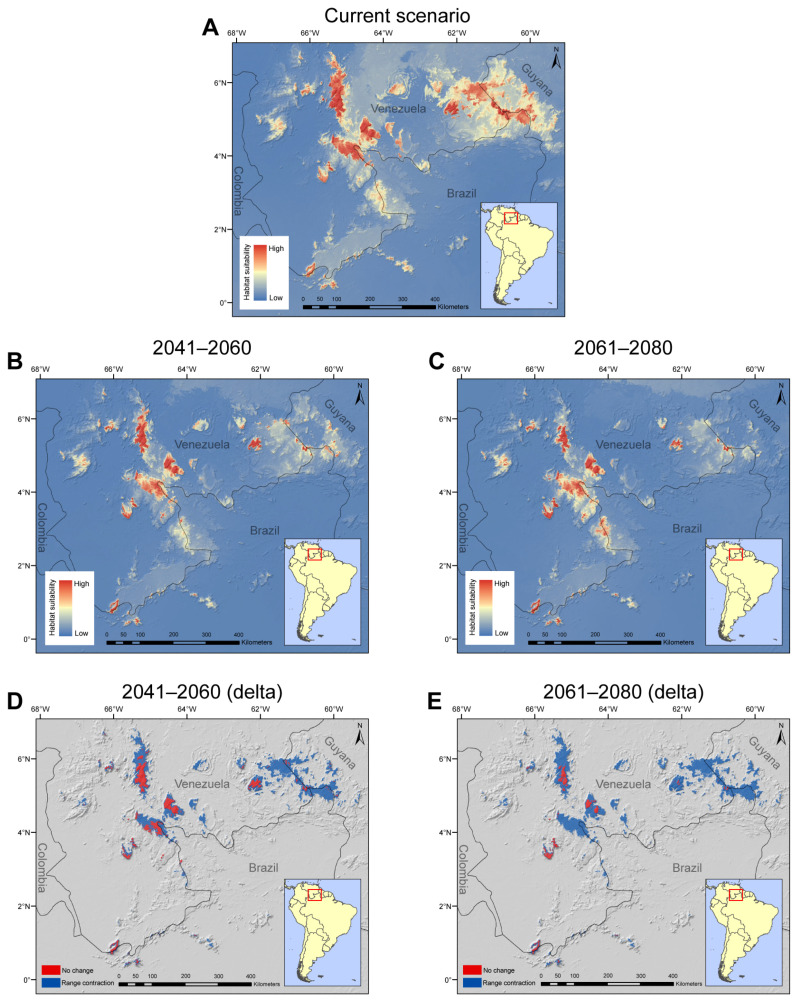
Modelled distribution of *Prosthechea jauana* for current and future projected bioclimatic data using MaxEnt. (**A**). Model for current bioclimatic data. (**B**). Model for future projected bioclimatic data in 2041–2060. (**C**). Model for future projected bioclimatic data in 2061–2080. (**D**,**E**). Range contraction of the suitable habitat area on the 2041–2060 and 2061–2080 models compared to the current conditions model, respectively.

The MaxEnt modeling predicted a slightly larger area than the current known distribution of *P. jauana* (Figure 3A). However, this is not entirely unexpected considering that the predicted suitable habitats are contiguous with the current distribution and represent similar mountain habitats where the species currently occur. The average AUC and the TSS values of the model were 0.91 and 0.65, respectively, indicating the good predictive performance of our model. Out of the five bioclimatic variables, mean annual temperature (Bio 1) and precipitation in the coldest quarter (Bio 19) had the strongest effect on the distribution of *P. jauana* (Appendix A; Table A1). Projecting the model into future climate scenarios revealed a significant loss in suitable habitats for *P. jauana*, with a reduction in area of ~79% by 2041–2060 and ~92% by 2061–2080 (Figure 3B–E). The future climate refugia of the species is observed to be in part of the Venezuelan Pantepui, and the suitable habitats in Brazil (including Serra do Aracá, the first record of the species in the country presented in this paper) are projected to experience a rapid reduction in the future.

## 3. Discussion

### 3.1. A Species Widely Distributed throughout a Sky Island Archipelago

Although *Prosthechea jauana* has been officially found (deposited voucher specimens) only on the Cerro Yaví, Meseta de Jaua (including Cerro Sarisariñama), and the Serra do Aracá, there is a record from the Auyán tepui, Dunsterville A99/A, cited as *Epidendrum fragrans* by Schweinfurth in Steyermark [22], which is also likely to be *Prosthechea jauana* (we used this record in our SDM). However, we could not find any voucher material (specimen or drawing) from the record to confirm its taxonomic identity. Unfortunately, the Dunsterville’s had a very peculiar system of annotations and botanical collections, where they frequently neglected common species. In a publication reporting the field expedition to Meseta de Jaua [23], they refer to *P. jauana* as an “odd-looking” and “depauperate form” of *Epidendrum fragrans*, which may explain why the annotation of the record Dunsterville A99/A in Auyán tepui has not a proper collection or associated illustration.

Nevertheless, considering the scarcity of records for the species combined with how spread across the Pantepui the known records are, it is presumable that *P. jauana* may occur in other tepuis, which is suggested in our SDM based on current bioclimatic conditions (Figure 3A). The model predicts occurrence of *P. jauana* in all Pantepui phytogeographical districts [24]. Therefore, it should be only matter of time and field work effort in this remote zone before new records of the species become reported from other tepuis.

### 3.2. The Relevance of Taxon-Focused Studies

The taxonomic complexity factor associated with the *Prosthechea fragrans* complex becomes evident as other orchid taxonomists also misidentified the material [Labiak et al. 5690 (RB)] as *P. fragrans*, which was followed and later used by Barbosa-Silva et al. [7]. Thus, the correct taxonomic identity of the material presented here, leading to the recognition of a further new record in the Brazilian flora and the southwards expansion of the species’ distribution, highlights the relevance of taxon-focused monograph studies on clarifying species delimitation and their geographic distribution. Similar recent efforts have also expanded the distribution of other orchid species in the Pantepui [25,26], and new species have been described as well [27].

### 3.3. Implications of Climate Change over a Pantepui Endemic Orchid

The projected future loss of suitable habitat for *P. jauana*, as revealed in the model, raises significant concerns in the face of impending climate change. The stark reduction in suitable habitat of nearly 78% by 2041–2060 and a staggering 91% by 2061–2080 underscore the urgency of addressing the impact of climate change on the species’ distribution. The loss of plant populations in the Pantepui is a reality for many endemic lineages [14] and *P. jauana* is one of many more species that may be severely affected by this scenario, as shown in the dramatic reduction in suitable habitats until 2080 according to our SDM’s future projection. In this sense, the lack of climatic refuges for plants is a serious issue that threatens many species. As climate change intensifies, traditional refuges in the Pantepui are becoming increasingly obsolete, and plant populations may struggle to adapt to changing conditions [18].

The threat to or loss of plant diversity has a ripple effect throughout the ecosystem, impacting several species that rely on these plants for shelter or as a host, altering the balance of ecological relationships [28]. Epiphytic plants are highly dependent on trees since they usually depend on a phorophyte for their establishment. However, the phorophytes are also often threatened by climate change and this can drive the loss of epiphyte diversity [29,30]. Addressing this problem will require a coordinated effort by conservationists, policymakers, and scientists to identify and protect habitats that may serve as refuges.

In an evaluation of the impacts of climate change over areas of global biodiversity importance, Manes et al. [31] suggest overall warming level thresholds based on IPCC data [32], where an increase in global surface air temperature (Gsat) to 1.5 °C above pre-industrial levels would have a relatively mild impact on biodiversity; 1.5–2 °C would have a moderate impact; 2–3 °C would have a high impact; and >3 °C is considered to have a very high impact on biodiversity. After their meta-analysis, the authors were able to conclude that endemic species will be more severely affected by climate change than introduced or non-endemic native species, especially those inhabiting island and mountain regions, where the extinction risk could be over six times higher than in mainland regions.

Conservation policies should consider prioritizing epiphytic plants from Pantepui. While many tepuis are currently within protected areas [24], global climate change poses a significant threat to these species in mountain ecosystems, suggesting that the protection of these areas alone may not be enough for their conservation [33]. Hence, efforts to conserve these species from Pantepui should be launched, planned, and realized in botanical gardens, germplasm banks, and other institutions offering ex situ conservation. Additionally, further efforts to better understand the ecology and biology of *P. jauana* may contribute to effective conservation strategies. Recent advances have been made regarding the effect of soil on plant composition in tepuis [16,34]; however, further progress is needed to understand the effects on epiphyte communities in the Pantepui region.

## 4. Materials and Methods

### 4.1. Study of Herbarium Materials

The new record was identified when revising the RB collection, but other relevant herbaria for the Pantepui and Amazonian flora were revised as well, such as AMES, BH, F, IAN, INPA, MG, MO, NY, US, and VEN. The morphological study of the specimens was carried out using a stereomicroscope and standard measurement tools. Specimens analyzed through high resolution images were measured using ImageJ version 1.51w [35]. A flower of the new record specimen was rehydrated, dissected, and mounted on a paper card for a detailed morphological description. All measurements of vegetative organs were based on dried material, except the flowers, which were rehydrated. The morphological terminology followed Radford et al. [36], Dressler [37], and Beentje [38].

Records without geographic coordinates data had their locality coordinates recovered using Google Earth Pro version 7.3.6 after the analysis of information present on the specimens’ labels. The distribution map (Figure 1) was produced using ArcGIS version 10.5 [39]. Preliminary conservation assessment followed the criteria and guidelines of the IUCN [40,41]. The extent of occurrence (EOO) and area of occurrence (AOO) were estimated using GeoCAT (http://geocat.kew.org/, accessed on 2 November 2023) [42], based on geographic data of the known occurrences. For the AOO estimation, a 2 km^2^ grid cell was adopted as recommended by IUCN [41].

### 4.2. Species Distribution Modeling

The maximum entropy (Maxent) approach is one of the most widely used ecological niche modeling tools for predicting species distributions [43,44,45,46]. Maxent is part of the machine learning method family, modeling potential species’ distribution using a set of environmental variables and presence-only georeferenced data [47]. It has been shown to outperform other algorithms, even when applied to small datasets [48,49], becoming one of the most popular methods used today. We implemented Maxent using version 3.4.4 of the software [50], with default settings. We used the minimum bounding rectangle covering all the occurrence points plus nearly 200 km buffer around the bounding rectangle to demarcate the modeling area for our analyses. Although this may represent an arbitrary decision prior to the analysis, it was based on the notably reduced sample size, looking to optimize the model’s performance. Nevertheless, our modeling area includes all mountain ranges that are contiguous with the current known distribution of *P. jauana* and may reflect locations where the species might have potential populations.

Due to the very small number of presence records (only 5 occurrence points), we used the jackknife (‘leave-one-out’) technique to model the distribution of *P. jauana*, an approach that has been found to be ideal for modeling species with few presence records [51]. Each observed locality was removed from the data set once, and a model was built using the remaining localities, yielding models equal to the total number of presence records (5 models in our case). The average of all five models was used to obtain the final prediction. Each model’s goodness-of-fit was evaluated using the average value of the area under the receiver operating characteristic curve (AUC) and true skill statistics (TSS) estimated from the five models. AUC values less than 0.7 indicate poor model performance, AUC values between 0.7 and 0.9 indicate moderate model performance, and AUC values greater than 0.9 indicate excellent model performance. Similarly, TSS values of 0.2–0.5 indicate a poor model, 0.6–0.8 indicate a good model, and greater than 0.8 indicate an excellent model [52]. We used ‘maximize test sensitivity plus specificity’ as a threshold criterion to obtain binary predictions from the model. The change in suitable habitats was calculated by overlaying the predicted future distribution over the predicted current distribution in ArcGIS 10.5.

Environmental data (19 bioclimatic variables) were obtained from the WorldClim database [53] at the spatial resolution of 30 arc seconds. However, we did not use all 19 variables for building models due to multicollinearity issues, since it may violate statistical assumptions and influence model predictions [54]. We removed strongly correlated (correlation coefficient > 0.7; or <−0.7) variables and retained biologically meaningful variables that had relatively high contribution in preliminary analysis. The resulting variable sets included 5 predictors (Appendix A; Table A1). In order to evaluate the change in future suitable habitat for *P. jauana*, we projected the model into future bioclimatic conditions. We obtained bioclimatic data for future conditions also from the WorldClim database, at the spatial resolution of 30 arc seconds, for two time periods: 2041–2060 and 2061–2080, based on the MIROC6 global climate model and SSP585 shared socio-economic pathway [55].

## 5. Conclusions

*Prosthechea jauana* is a poorly known orchid species endemic to the Pantepui region of the Guiana Shield Highlands, with just a few materials deposited in biological collections. The recent discovery of *P. jauana* in Serra do Aracá, a tepui on the northern border of the Brazilian Amazon, previously unreported in the literature, highlights the importance of continued exploration and research in the region, as well as the relevance of group-focused taxonomic studies. Nevertheless, this discovery also raises concerns about the conservation status of the species and the Pantepui endemic flora in the climatic future. SDM of future bioclimatic scenarios suggests a dramatic reduction in suitable areas for the species’ occurrence.

## Data Availability

All generated data are included in this article.

## Appendix A

**Table A1:** Bioclimatic variables used and their relative contribution to generating the species’ distribution model for *Prosthechea jauana*.

Bioclimatic Variable	Rate of Contribution (%)
Mean annual temperature (Bio 1)	75.9
Temperature seasonality (Bio 4)	3.7
Annual range of temperature (Bio 7)	2.2
Annual precipitation (Bio 12)	0.4
Precipitation of coldest quarter (Bio 19)	17.8
